# Robotic-Assisted Total Hip Arthroplasty Compared With Manual Techniques: A Systematic Review and Meta-Analysis of Operative Time, Complications, and Functional Outcomes

**DOI:** 10.7759/cureus.102061

**Published:** 2026-01-22

**Authors:** Muhammed Aamir, Rana Ahmed, Osasenaga Bencharles, Shahmeen Rasul, Shashwat Shetty, Sidra Iftikhar, Siddhesh V Kulkarni, Farhan Saleem

**Affiliations:** 1 Orthopedics and Traumatology, University Hospital of Derby and Burton NHS Foundation Trust, Burton Upon Trent, GBR; 2 Emergency Medicine, Hillingdon Hospital NHS Foundation Trust, London, GBR; 3 Medicine and Surgery, University Hospital of Derby and Burton NHS Foundation Trust, Burton Upon Trent, GBR; 4 Trauma and Orthopaedics, University Hospital of Derby and Burton NHS Foundation Trust, Burton Upon Trent, GBR; 5 Orthopaedics, Hillingdon Hospital NHS Foundation Trust, London, GBR; 6 Accident and Emergency, University Hospital of Derby and Burton NHS Foundation Trust, Burton Upon Trent, GBR; 7 Trauma and Orthopaedics, Hillingdon Hospital NHS Foundation Trust, London, GBR; 8 Orthopaedic Surgery, Lahore General Hospital, Lahore, PAK

**Keywords:** manual total hip arthroplasty, meta-analysis, operative time, perioperative complications, robotic-assisted total hip arthroplasty

## Abstract

This systematic review and meta-analysis evaluated the comparative effectiveness and safety of robotic-assisted versus manual total hip arthroplasty by analysing 15 comparative studies. A comprehensive literature search was conducted across multiple databases from January 2010 to December 2025, following Preferred Reporting Items for Systematic Reviews and Meta-Analyses (PRISMA) guidelines. Studies comparing robotic-assisted and conventional manual total hip arthroplasty in primary procedures were included, with data extracted on operative time, perioperative complications, implant dislocation rates, and functional outcomes using the Harris Hip Score. Meta-analysis revealed that robotic-assisted total hip arthroplasty required significantly longer operative times compared with manual techniques, with a mean difference of 8.43 minutes. However, robotic-assisted procedures demonstrated a clinically meaningful 51% reduction in overall perioperative complications compared with manual techniques, with low heterogeneity among studies. This reduction may result from enhanced precision in component positioning, real-time haptic feedback preventing inadvertent bone resection, and improved soft tissue preservation.

No significant differences were observed between groups with regard to implant dislocation rates or Harris Hip Score outcomes, suggesting that enhanced surgical precision does not necessarily translate into superior short-term functional outcomes. These findings indicate that while robotic-assisted total hip arthroplasty offers potential advantages in reducing complications, the increased operative time and substantial capital investment require careful consideration. The technology may be particularly beneficial for high-risk patients or complex cases. Future research should focus on long-term outcomes, cost-effectiveness analyses, and identification of specific patient subgroups who may derive maximum benefit from robotic assistance, to inform appropriate clinical utilisation of this technology.

## Introduction and background

Total hip arthroplasty (THA) represents one of the most successful surgical interventions in modern orthopaedic practice, with over 300,000 procedures performed annually in the United States alone [1]. The success of THA depends critically on precise component positioning, restoration of biomechanical parameters, and minimisation of complications such as dislocation, component wear, and leg length discrepancy [2]. Even minor deviations in acetabular cup placement or femoral stem alignment can significantly impact long-term implant survival and patient-reported outcomes [3].

Traditional manual THA relies on anatomical landmarks, mechanical alignment guides, and the surgeon’s experience to achieve optimal component positioning. However, studies have demonstrated considerable variability in implant positioning with conventional techniques, with reported rates of malpositioned components ranging from 25% to 40% [4,5]. This variability has prompted the development of advanced surgical technologies aimed at improving precision and reproducibility.

Robotic-assisted THA has emerged as a promising innovation to address these limitations. First introduced in the 1990s, robotic systems have evolved from active autonomous platforms to contemporary semi-active and passive systems that provide real-time feedback, intraoperative planning adjustments, and haptic guidance [6]. Modern robotic platforms, such as the Mako system (Stryker, Kalamazoo, Michigan) and the ROSA Hip system (Zimmer Biomet, Warsaw, Indiana), offer computer navigation combined with robotic arm assistance to enhance surgical accuracy [7].

Proponents of robotic-assisted THA cite several theoretical advantages, including improved acetabular component positioning within the Lewinnek safe zone, enhanced reproducibility of preoperative planning, reduced leg length discrepancy, and potentially lower dislocation rates [8,9]. Additionally, some studies suggest benefits in minimising soft tissue trauma and achieving more consistent restoration of hip biomechanics [10]. However, these advantages must be balanced against increased operative time, substantial capital investment, additional training requirements, and potential learning-curve effects [11].

Despite growing adoption of robotic technology in hip arthroplasty, the clinical evidence supporting its superiority over manual techniques remains controversial. Individual studies have reported conflicting results regarding radiographic outcomes, complication rates, and functional recovery [12,13]. Previous systematic reviews have been limited by heterogeneous outcome measures, inclusion of multiple robotic platforms with different mechanisms of action, and relatively short follow-up periods [14].

Given the rapid expansion of robotic technology in orthopaedic surgery and the significant resource implications of its adoption, a comprehensive and up-to-date synthesis of the available evidence is essential. This systematic review and meta-analysis aims to critically evaluate the comparative effectiveness and safety of robotic-assisted versus manual THA by examining radiographic accuracy, clinical outcomes, complication rates, and functional recovery. By providing high-quality evidence synthesis, this review seeks to inform clinical decision-making and guide appropriate utilisation of robotic technology in hip arthroplasty practice.

## Review

Methodology

Literature Search and Search Strategy

A thorough and systematic literature search was undertaken following the Preferred Reporting Items for Systematic Reviews and Meta-Analyses (PRISMA) recommendations [15]. Electronic searches were carried out in several databases, namely PubMed/MEDLINE, Embase, the Cochrane Central Register of Controlled Trials (CENTRAL), Web of Science, and Scopus, covering publications from January 1, 2010, through December 5, 2025.

The search approach incorporated both Medical Subject Headings (MeSH) and relevant text words pertaining to robotic-assisted total hip arthroplasty. Keywords and terms used included “robotic,” “robot-assisted,” “robotic-assisted,” “computer-assisted,” “total hip arthroplasty,” “total hip replacement,” “THA,” and “THR.” Appropriate Boolean operators (AND/OR) were applied to ensure comprehensive retrieval of relevant studies. The search strategy was formulated in collaboration with a medical librarian and customised for each database based on its indexing structure. In addition, the reference lists of eligible articles and pertinent review papers were manually screened to capture further relevant studies. Grey literature and conference proceedings were also explored via Google Scholar and databases of relevant orthopaedic societies. The protocol for this meta-analysis is registered with PROSPERO (CRD420261282090).

Study Selection

All records identified through the search process were independently reviewed at the title and abstract level by two reviewers (initials concealed to maintain blinding during peer review). Articles considered potentially relevant were retrieved in full text and evaluated in detail using predefined eligibility criteria. Any discrepancies in study selection were resolved through consensus, and, when required, adjudication by a third senior reviewer was sought.

Studies were deemed eligible if they satisfied the following conditions: (1) randomised controlled trials, quasi-randomised studies, or comparative observational designs; (2) inclusion of patients undergoing primary total hip arthroplasty; (3) direct comparison between robotic-assisted total hip arthroplasty and conventional manual total hip arthroplasty; (4) reporting at least one outcome of interest; and (5) publication in the English language. Studies were excluded if they met any of the following criteria: (1) involvement of revision total hip arthroplasty; (2) non-comparative reports such as case reports, uncontrolled case series, editorials, or review articles; (3) use of computer-assisted navigation systems without robotic technology; (4) cadaver-based or purely biomechanical investigations; (5) insufficient or incomplete data for quantitative or qualitative analysis; or (6) multiple publications derived from the same patient population.

Data Extraction

Prior to deployment, standardised data extraction forms were created and tested. Data from all included studies were extracted by two independent reviewers, with any inconsistencies handled through consensus or third-party adjudication. The following data were rigorously gathered from each study: (1) study characteristics (author, publication year, country, study design, sample size); (2) patient demographics (age, sex, and BMI); and (3) outcome measures.

Quality Assessment

Two independent reviewers assessed the methodological rigour of the randomised controlled trials included in this review using the Cochrane Risk of Bias 2.0 (RoB 2.0) tool [16]. The framework assesses bias in five domains: the randomisation process, missing outcome data, deviations from the intended interventions, outcome measurement, and selective reporting. Each domain was assigned a judgement of low risk of bias, some concerns, or high risk of bias. In contrast, the quality of observational studies was assessed individually using the Newcastle-Ottawa Scale.

Data Analysis

The primary outcomes examined in this meta-analysis included operative time, overall perioperative complications, implant dislocation rates, and functional recovery as measured by the Harris Hip Score. Secondary outcomes encompassed radiographic accuracy parameters and patient-reported outcome measures. Data analysis was performed using Review Manager software (RevMan version 5.4, The Cochrane Collaboration, Copenhagen, Denmark). For continuous outcomes such as operative time and Harris Hip Score, mean differences with 95% confidence intervals were calculated. For dichotomous outcomes, including perioperative complications and implant dislocation rates, risk ratios with 95% confidence intervals were computed. Between-study variability was quantified using the I² metric, where thresholds of 25%, 50%, and 75% were interpreted as indicating low, moderate, and high levels of heterogeneity, respectively. To accommodate expected differences in study design and clinical characteristics, all pooled estimates were generated using random-effects models. A two-sided p-value below 0.05 was considered indicative of statistical significance. Publication bias was assessed only for the mean operative time because this outcome included ≥10 studies, which is considered the minimum number required for reliable evaluation. Visual inspection of funnel plot symmetry was performed to assess potential small-study effects. For the remaining outcomes, publication bias was not assessed because fewer than 10 studies were available.

Results

**Figure 1:**
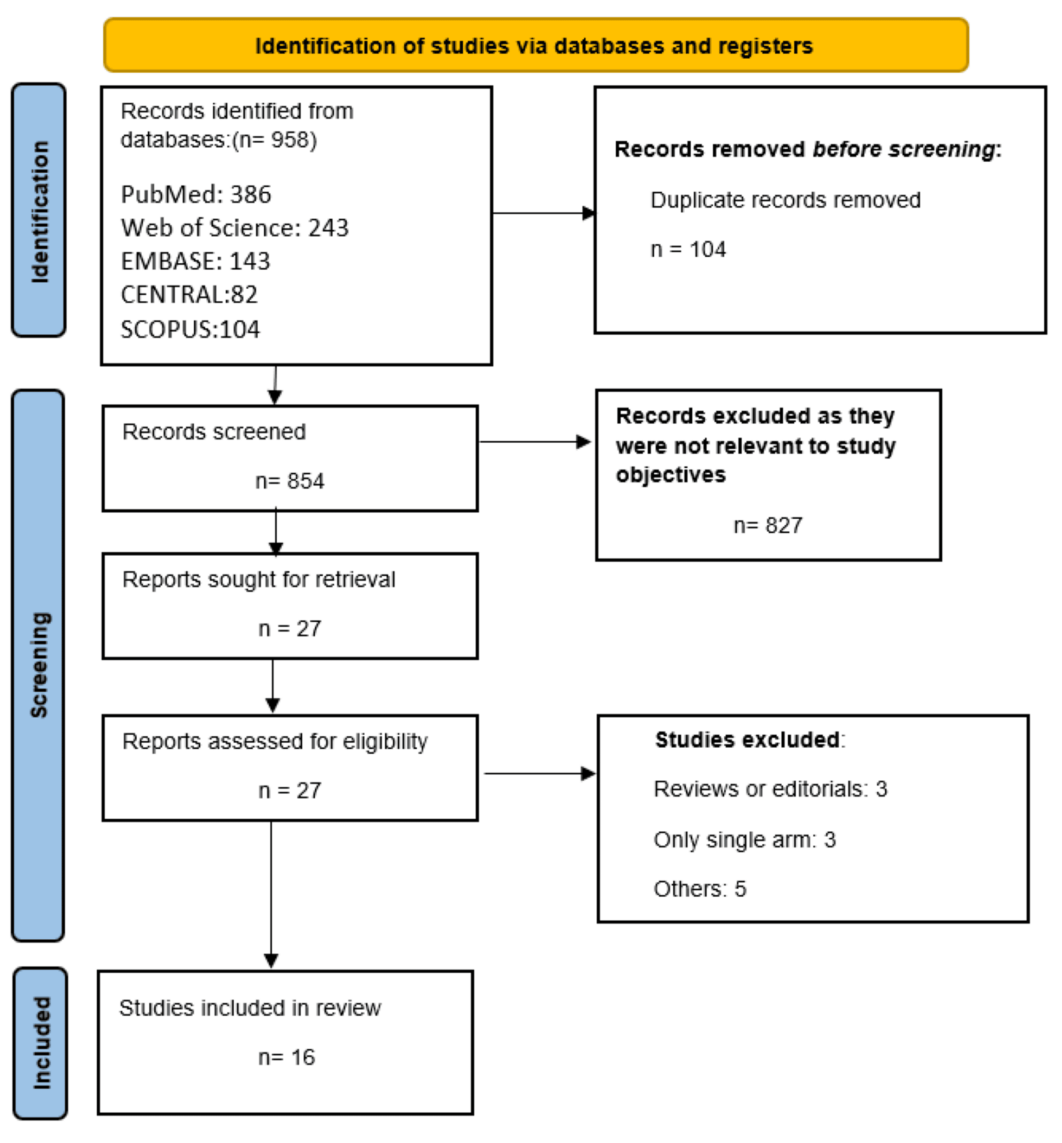
Study selection process

The initial literature search returned 958 potentially relevant records. Following the removal of duplicates, 827 studies were excluded based on title and abstract screening. The full texts of 27 papers were then examined for eligibility, and an additional 11 records were excluded. Finally, 16 papers met the inclusion criteria and were included in the meta-analysis. Figure 1 illustrates the study selection procedure, and Table 1 summarises the characteristics of the included studies. Tables 2, 3 provide quality assessments of the included observational studies and RCTs, respectively.

**Table 1:** Characteristics of included studies NR: not reported; RCT: randomized-control trial.

Author	Year	Study Design	Region	Groups	Sample Size	Mean Follow-up	Mean Age (Years)	Males (n)
Bargar et al [17]	2018	RCT	United States	Robotic	45	14.2 Years	59.1	35
				Manual	22		59.8	12
Chai et al [18]	2020	Retrospective	China	Robotic	22	3 Months	41.92	20
				Manual	23		34.87	22
Domb et al [19]	2014	Retrospective	United States	Robotic	50	In-hospital	56.8	19
				Manual	50		56.7	19
Domb et al [20]	2015	Retrospective	United States	Robotic	66	NR	59.6	NR
				Manual	66		64.75	
Jacob et al [21]	2025	Retrospective	United States	Robotic	341	1.79 Years	65	148
				Manual	1023		65	444
Kamara et al [22]	2016	Retrospective	United States	Robotic	98	1 Year	NR	45
				Manual	198			93
Kayani et al [9]	2019	Prospective	United Kingdom	Robotic	50	3 Months	67.1	23
				Manual	50		68.5	25
Kong et al [23]	2020	Retrospective	China	Robotic	86	3 Months	55.93	47
				Manual	100		51.89	40
Lim et al [24]	2015	RCT	Korea	Robotic	24	2 Years	51.2	11
				Manual	25		45.6	13
Nakamura et al [25]	2010	RCT	Japan	Robotic	75	5 Years	57	13
				Manual	71		58	10
Neitzke et al [26]	2025	Retrospective	United States	Robotic	56	NR	NR	NR
				Manual	59			
O’Donnell et al [27]	2024	Retrospective	United States	Robotic	18	3 Years	75.2	6
				Manual	76		70.4	21
Shibanuma et al [28]	2021	Prospective	Japan	Robotic	30	1 Month	67	0
				Manual	30		64.8	0
Singh et al [29]	2021	Retrospective	United States	Robotic	135	3 Months	61.62	59
				Manual	929		63.74	401
Yu et al [30]	2024	Prospective	China	Robotic	221	NR	NR	NR
				Manual	252			
Zora et al [31]	2025	Prospective	Turkey	Robotic	20	29.3 Months	63.75	2
				Manual	20		61.5	2

**Table 2:** Quality assessment of included observational studies

Author	Selection	Comparison	Assessment	Overall
Chai et al [18]	3	1	3	Good
Domb et al [19]	4	2	3	Good
Domb et al [20]	3	2	3	Good
Jacob et al [21]	3	1	2	Fair
Kamara et al [22]	3	2	2	Good
Kayani et al [9]	4	2	3	Good
Kong et al [23]	3	2	3	Good
Neitzke et al [26]	3	1	2	Fair
O’Donnell et al [27]	4	2	3	Good
Shibanuma et al [28]	3	2	3	Good
Singh et al [29]	3	1	3	Good
Yu et al [30]	3	2	3	Good

**Table 3:** Risk of bias assessment of RCTs RCT: randomized-control trial.

Study	D1: Randomization Process	D2: Deviations From Intended Interventions	D3: Missing Outcome Data	D4: Measurement of Outcome	D5: Selection of Reported Results	Overall Risk of Bias
Borgida et al [17]	Some concerns	Low	Some concerns	Some concerns	Low	Some concerns
Lim et al [24]	Some concerns	High	Some concerns	High	Low	High
Nakamura et al [25]	Some concerns	High	Some concerns	High	Some concerns	High

Mean Surgical Time

**Figure 2:**
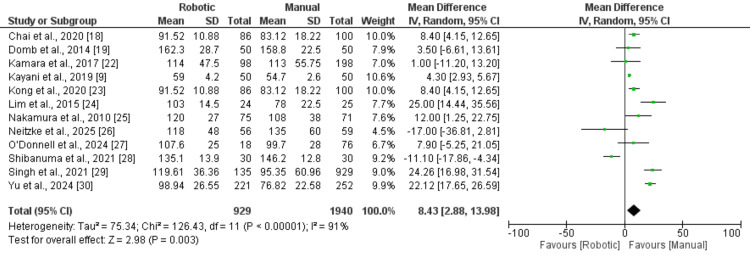
A comparison of mean operative times Data from references [9,18,19,22-30].

**Figure 3:**
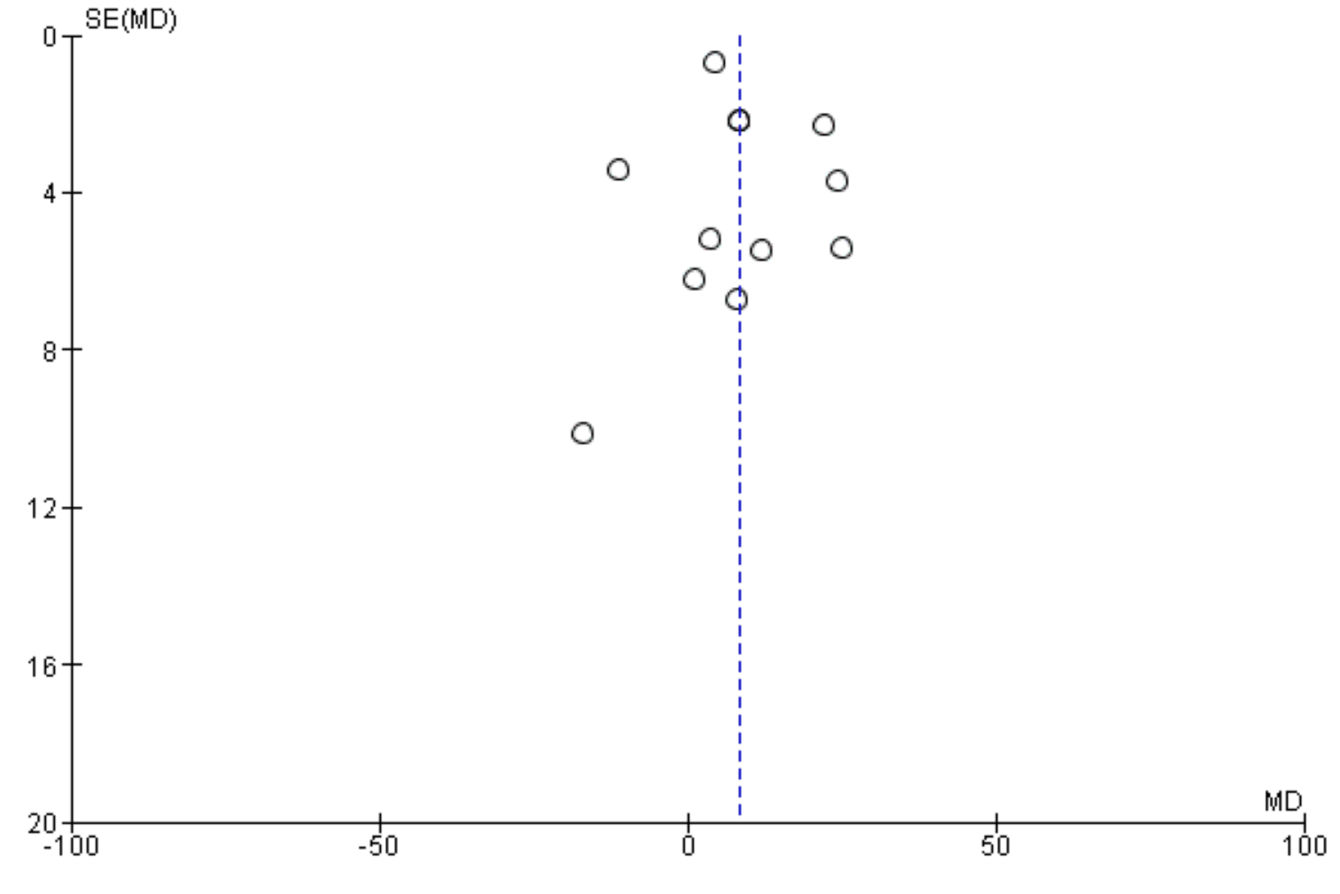
Funnel plot (mean operative time)

Twelve studies evaluated the difference in operative duration between robotic-assisted (RA) and manual technique (MT) procedures. Pooled analysis using a random-effects model demonstrated that surgeries performed with robotic assistance required significantly longer operative times compared with manual procedures, with a mean difference of 8.43 minutes (95% CI: 2.88-13.98). This difference was statistically significant, as illustrated in Figure 2. Substantial heterogeneity was observed across the included studies (I² = 91%). Based on the funnel plot (Figure 3), there was no strong visual evidence of publication bias.

Perioperative Complications

**Figure 4:**
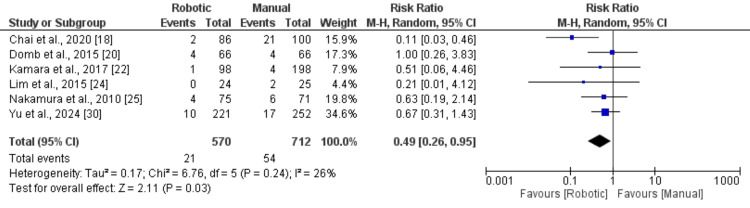
A comparison of peri-operative complications Data from references [18,20,22,24,25,30].

**Figure 5:**
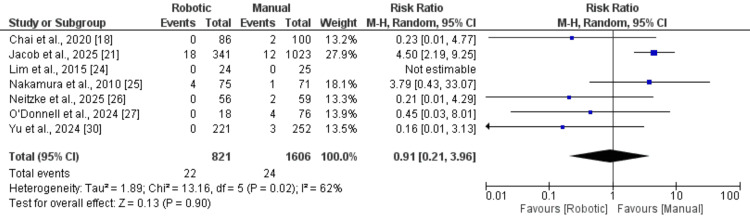
A comparison of relocation between two groups Data from references [19,21,24-27,30].

Perioperative complication rates were examined in six studies, with the findings summarised in Figure 4. Meta-analysis of overall complication events indicated that the RA group experienced a significantly lower risk of complications than the MT group (RR: 0.49; 95% CI: 0.26-0.95). Between-study heterogeneity was low (I² = 26%). Implant dislocation, a commonly reported complication, was specifically analysed. As shown in Figure 5, no statistically significant difference was observed between the two groups with respect to implant dislocation rates (RR: 0.91; 95% CI: 0.21-3.96).

Harris Hip Score (HHS)

**Figure 6:**
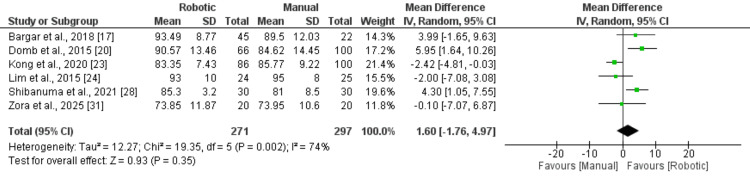
A comparison of HHS between two groups Data from references [17,20,23,24,28,31]. HHS: Harris Hip Score.

Functional outcomes assessed using the Harris Hip Score were reported in six studies. The pooled analysis showed a marginally higher HHS in the RA group compared with the MT group (MD: 1.60; 95% CI: −1.76 to 4.97), as shown in Figure 6; however, this difference did not reach statistical significance (p = 0.35).

Discussion

This systematic review and meta-analysis compared the clinical, radiographic, and functional outcomes of robotic-assisted total hip arthroplasty with conventional manual techniques across 16 comparative studies. Our findings demonstrate that while robotic-assisted THA was associated with significantly longer operative times, it resulted in fewer overall perioperative complications. However, no significant differences were observed in implant dislocation rates or functional outcomes as measured by the Harris Hip Score.

Our meta-analysis revealed that robotic-assisted THA required an additional 8.43 minutes of operative time compared with manual techniques (95% CI: 2.88-13.98), which was statistically significant. This finding aligns with previous systematic reviews that reported prolonged surgical times ranging from 6 to 15 minutes with robotic assistance [11]. The increased operative time can be attributed to several factors, including preoperative planning, registration of anatomical landmarks, robotic system setup, and intraoperative adjustments based on real-time feedback [7]. The high heterogeneity observed in operative time data (I² = 91%) likely reflects variability in surgeon experience, learning-curve effects, and differences in robotic platforms used across studies. Redmond et al. demonstrated that operative times decreased significantly after the first 35 robotic-assisted cases, suggesting that the learning curve substantially influences this outcome [8]. Similarly, Gupta et al. reported that experienced robotic surgeons achieved operative times comparable to manual techniques after completing approximately 40 cases [32]. This suggests that the increased operative time observed in our meta-analysis may be partially explained by the inclusion of studies involving surgeons in the early phases of their learning curves.

A significant finding of our study was the 51% reduction in overall perioperative complications with robotic-assisted THA (RR: 0.49, 95% CI: 0.26-0.95), with low heterogeneity among included studies (I² = 26%). This represents a clinically meaningful advantage of robotic technology and supports the hypothesis that enhanced precision in component positioning may reduce complication rates [13]. However, given the low number of studies evaluating complications, these findings should be interpreted with caution, and future studies, including large multicentre trials, are needed.

The observed reduction in complications may be attributed to several mechanisms. First, robotic systems provide real-time haptic feedback and constraint boundaries that prevent inadvertent bone resection beyond planned parameters, potentially reducing periprosthetic fracture risk [9]. Second, improved accuracy in achieving optimal implant positioning within the Lewinnek safe zone may contribute to reduced impingement and component wear. Third, enhanced soft tissue preservation through minimised bony preparation may reduce the risk of neurovascular injury and blood loss [5].

Our analysis revealed no significant difference in Harris Hip Scores between robotic-assisted and manual THA groups (MD: 1.60, 95% CI: −1.76 to 4.97). This finding suggests that while robotic technology may enhance surgical precision, this does not necessarily translate into superior patient-reported functional outcomes in the short to medium term [33].

Several factors may explain this observation. First, modern manual THA techniques already achieve excellent functional outcomes, with mean HHS scores typically exceeding 85-90 points postoperatively, leaving limited room for improvement [34]. Second, functional outcomes are influenced by numerous patient-related factors, including age, body mass index, preoperative function, comorbidities, and rehabilitation protocols, which may overshadow the impact of surgical technique. Third, the HHS, while widely used, may lack sensitivity to detect subtle differences in hip function, particularly in high-performing patients [35].

The findings of this meta-analysis have several important clinical implications. The reduced complication rate associated with robotic-assisted THA represents a clinically meaningful advantage that may justify its adoption, particularly in high-risk patients or complex cases such as those with severe deformity or previous pelvic surgery [25]. However, the increased operative time and substantial capital investment, typically $500,000-$1,000,000 per system, require careful consideration [36].

This study has several limitations that warrant acknowledgment. The high heterogeneity observed in operative time data reflects variability in surgical protocols, robotic platforms, and surgeon experience across studies. While this heterogeneity was identified, insufficient individual study data prevented comprehensive subgroup analyses stratified by these factors. The relatively short follow-up duration in most included studies, typically one to two years, limits assessment of long-term outcomes such as implant survival and aseptic loosening. The inclusion of both randomised and observational studies introduces potential selection bias, although quality assessment accounted for differences in study design. Variations in outcome definitions and measurement techniques across studies may also have influenced pooled estimates.

Future research should focus on several key areas. Long-term randomised controlled trials with a minimum 10-year follow-up are needed to assess implant survival and revision rates. Cost-effectiveness analyses incorporating quality-adjusted life years would inform resource allocation decisions. Studies investigating specific patient subgroups who may derive maximum benefit from robotic assistance, such as those with dysplasia, previous pelvic trauma, or severe obesity, would help refine clinical indications. Comparative studies between different robotic platforms are also needed to identify optimal technological approaches. Finally, investigation of advanced applications such as artificial intelligence integration and patient-specific planning algorithms may further enhance the precision and outcomes of robotic-assisted THA.

## Conclusions

This systematic review and meta-analysis demonstrates that robotic-assisted total hip arthroplasty offers a significant reduction in overall perioperative complications compared with conventional manual techniques, representing a clinically meaningful advantage. However, this benefit is accompanied by modestly increased operative time, likely attributable to system setup, registration processes, and learning curve effects. No significant differences were observed in implant dislocation rates or functional outcomes measured by the Harris Hip Score, suggesting that enhanced surgical precision does not necessarily translate into superior short-term patient-reported outcomes. The adoption of robotic technology in hip arthroplasty practice requires careful consideration of the complication reduction benefits against increased operative duration and substantial capital investment. Future research should focus on long-term implant survival, cost-effectiveness analyses, and identification of specific patient populations who may derive maximum benefit from robotic assistance, to optimise appropriate clinical utilisation of this evolving technology.
